# Indole hydrazide compound ZJQ-24 inhibits angiogenesis and induces apoptosis cell death through abrogation of AKT/mTOR pathway in hepatocellular carcinoma

**DOI:** 10.1038/s41419-020-03108-2

**Published:** 2020-10-28

**Authors:** Jing Liu, Ying Liu, Jianqiang Zhang, Dan Liu, Yafeng Bao, Tianxing Chen, Tao Tang, Jun Lin, Ying Luo, Yi Jin, Jihong Zhang

**Affiliations:** 1 Laboratory of Molecular Genetics of Aging and Tumor, Medical School, Kunming University of Science and Technology, Kunming, 650500 P.R. China; 2 Key Laboratory of Medicinal Chemistry for Natural Resource, Ministry of Education, School of Chemical Science and Technology, Yunnan University, Kunming, 650091 P.R. China; 3 College of Biology and Chemistry, Key Laboratory of Subtropical Medicinal Edible Resources Development and Utilization in Yunnan Province, Puer University, Puer, 665000 Yunnan China; 4 Pathology Department, The First People’s Hospital of Yunnan Province, Kunming, 650032 P.R. China; 5 Guizhou Provincial Key Laboratory of Pathogenesis & Drug Development on Common Chronic Diseases, School of Basic Medicine, Guizhou Medical University, Guiyang, Guizhou 550000 China

**Keywords:** Tumour angiogenesis, Target identification

## Abstract

Angiogenesis and the activation of AKT/mTOR pathway are crucial for hepatocarcinoma development and progression, the activation of mTORC1/2 and relevant substrates have been confirmed in clinical hepatocarcinoma samples. Therefore, AKT/mTOR pathway represents the major targets for anti-cancer drugs development. Here, we investigated the anti-proliferative activity and mechanisms of ZJQ-24 in hepatocellular carcinoma, both in vivo and in vitro. A hepatocellular carcinoma xenograft model showed that ZJQ-24 significantly inhibited tumor growth with few side effects. MTT assays, flow cytometric analysis, Western blotting and immunohistochemistry identified that ZJQ-24 effectively suppressed hepatocellular carcinoma cell proliferation via G_2_/M phase arrest and caspase-dependent apoptosis but had no cytotoxic on normal cells. Furthermore, ZJQ-24 significantly blocked AKT/mTOR signaling by down-regulation of mTORC1 molecules, including phospho-p70S6K (Thr^389^) and phospho-4EBP-1 (Ser65, Thr^37/46^, Thr^70^) and phospho-AKT (Ser^473^) in HCC cells. It is very important that the ZJQ-24 did not induce the mTORC1-depdent PI3K/Akt feedback activation through JNK excitation. Moreover, ZJQ-24 inhibited the cap-dependent translation initiation by impairing the assembly of the eIF4E/eIF4G complex. Immunohistochemistry further confirmed ZJQ-24 inhibited the tumor growth through suppression of VEGF and AKT/mTOR pathways in vivo. Thus, the present study is the first to illustrate that ZJQ-24 triggers antiangiogenic activity and apoptosis via inhibiting the AKT/mTOR pathway in hepatocellular carcinoma cells, providing basic scientific evidence that ZJQ-24 shows great potential function as inhibitor of angiogenesis and tumor growth in hepatocellular carcinoma.

## Introduction

Hepatocellular carcinoma (HCC) is the fifth most common human cancer worldwide and the incidence of HCC has been increasing rapidly, particularly in China^1^. Although significant progresses in diagnosis and treatment of HCC (including target therapy) has been made, few drugs have been approved effective for HCC^2^. Not to mention almost all of them have side effects and quickly patients drug resistance^3^. Identification of novel molecular therapeutic targets and development of novel treatments are critical for HCC.

HCC is highly vascular tumor with a strong angiogenesis-inducing ability^4^. It has been reported that the expression of vascular endothelial growth factor (VEGF) was increased in HCC tumors and patients sera^5^, and high level of VEGF is associated with poor prognosis^6^. Therefore, VEGF and its downstream signaling might be a potential target for HCC treatment.

Besides VEGF activity, the mammalian target of rapamycin (mTOR) signaling pathway also plays a central role in regulation of cell proliferation, migration, survival, and angiogenesis, which represents a promising target with respect to its frequent dysregulation in HCC. Aberrant mTOR signaling activation is correlated with poor outcome of HCC^7^. mTOR, a highly conserved serine/threonine kinase is often detected in cancers and is regulated by various upstream regulators such as, PI3K and AKT^8,9^. It exists in two functionally distinct complexes: mTOR complex 1 (mTORC1) and mTOR complex 2 (mTORC2)^10^. In particular, the raptor-containing mTORC1 regulates mRNA translation and cell growth via phosphorylation of the eukaryotic initiation factor 4E binding proteins 4EBP-1 and the ribosomal protein S6 kinases (p70S6K). In addition, the rictor-containing mTORC2 modulates cell motility by phosphorylation of the AKT on Ser^473^, regulating AKT-mediated survival signaling^11,12^. Therefore, mTOR is an active target for drug discovery. Rapamycin and its derivatives have shown promising activity in a wide range of cancers^13^. However, rapamycin treatment can increase mTORC1-depdent PI3K/Akt feedback activation, the tolerance of some cancers to these agents has been reported^14^. To overcome this problem, multiple ATP-competitive mTOR kinase domain inhibitors that target both mTORC1 and mTORC2 have been developed (e.g., OSI-027 and AZD3147)^15,16^. But the potential activity of these agents against HCC has not been reported.

In addition, mTOR substrates are also used as potential drug targets^17^. Eukaryotic initiation factor (eIF) 4E, the mRNA 5’ cap-binding protein, is viewed as the end point target of the PI3K/AKT/mTOR axis and regulates mRNA translation initiation. The activity of eIF4E is repressed by 4EBP-1, which shows strong anti-tumor potential^18^. In normal cells, the hypophosphorylated forms of 4EBP-1 bind to eIF4E to inhibit the interaction of eIF4E and eIF4G, thus impairing cap-dependent translation^19^. Conversely, in cells with strong mTOR signaling, such as tumor cells, 4EBP-1 becomes hyper-phosphorylated and releases eIF4E to interact with eIF4G and assemble in to the eIF4F complex^20^. Then, the mitogen-activated protein kinases (MAPK)-interacting protein kinases MNK, which are activated by Erk, will phosphorylate the eIF4E on a unique site (Ser209, human sequence)^21^. Several recent reports demonstrated that dysregulated translation is omnipresent in human cancer^22^. In recent study, we originally synthesized a novel series of indole-2-carbohydrazide compounds as VEGFR2 tyrosine kinase inhibitor and evaluated for cytotoxicity in tumor cells and antiangiogenic effects in HUVECs^23^. However, the anti-hepatocellular carcinoma effects and functional mechanism of ZJQ-24, an active compound, remain unknown.

**Fig. 1:**
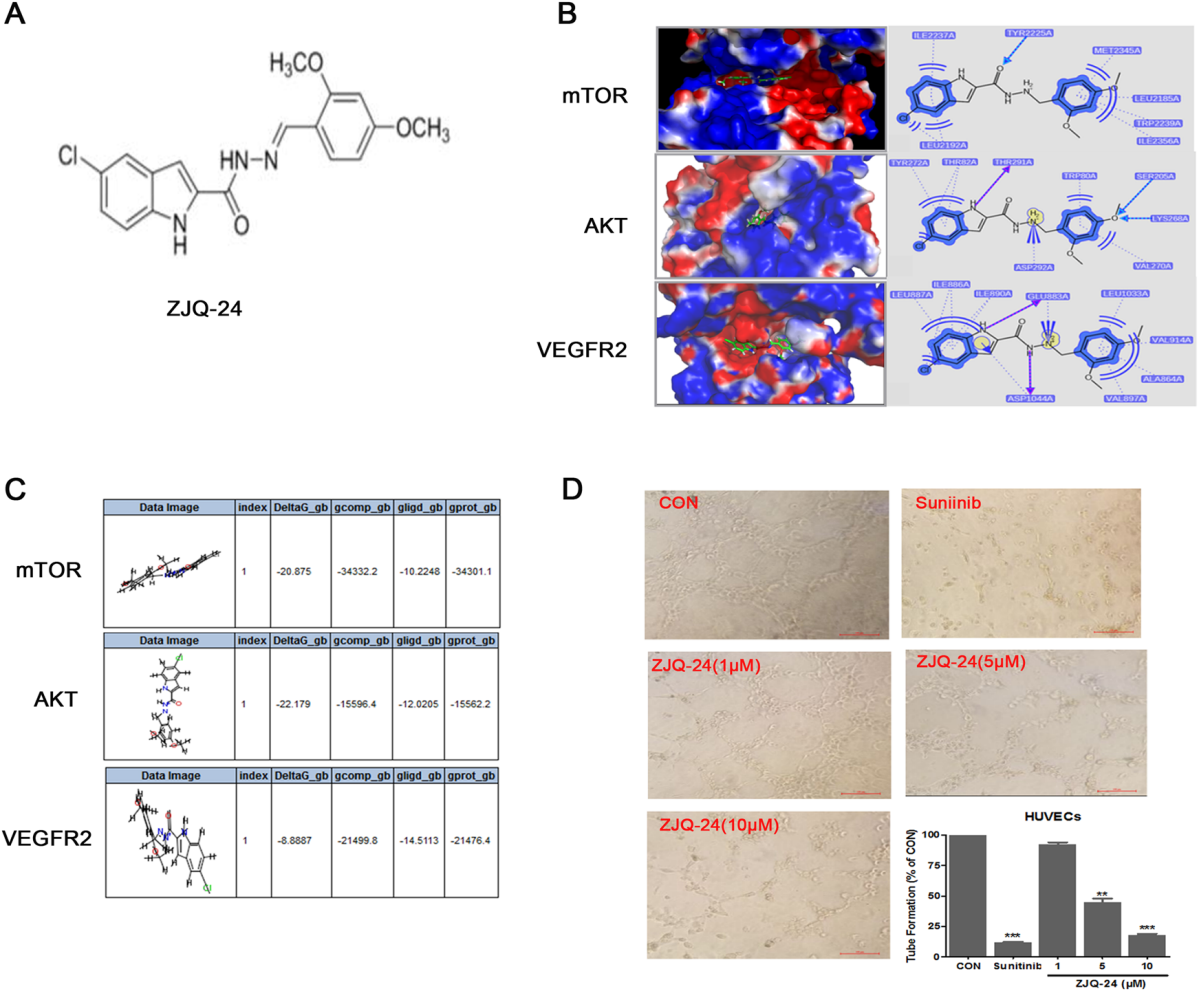
Docking study and anti- angiogenesis effect of ZJQ-24. **A** Chemical structure of ZJQ-24. **B** Compound ZJQ-24 docked into mTOR based on PDB code 4JT6, AKT based on PDB code 3O96. **C** The binding energies between ZJQ24 and proteins (mTOR, AKT and VEGFR2). **D** Tube formation inhibition. HUVECs were seeded on the surface of the Matrigel and treated with or without ZJQ-24. After incubation for 6 h, capillary tube formation was examined using an inverted microscope. *P < 0.05, **P < 0.01 *vs*. control group.

In the present study, we demonstrated the anti-hepatocellular carcinoma activity ZJQ-24 (5-chloro-*N*’-(2,4-dimethoxybenzylidene)−1*H*-indole-2-carbohydrazide) (Fig. 1A) for the first time and illustrated its potential mechanisms of action, involving angiogenesis inhibition, G2/M cycle arrest and cell apoptosis. In addition, ZJQ-24 significantly inhibits the AKT/mTOR pathway resulting in the suppression of cap-dependent translation initiation by impairing the eIF4E/eIF4G complex in HepG2 cells. We further showed that ZJQ-24 reduces the tumor growth in a HepG2 xenograft model through suppression of VEGF and AKT/mTOR pathways.

## Materials and methods

### Chemicals

ZJQ-24 compound was identified and provided by the Key Laboratory of Medicinal Chemistry for Natural Resource (Yunnan University)^23^. The compound was prepared as 100 mM stock solutions in DMSO and aliquots stored at −20 °C, protected from light. Reagents, unless specified otherwise, were purchased from Sigma-Aldrich Ltd (China).

### Tube formation assay

The effect of ZJQ-24 on the ability of HUVECs to form tube structures on Matrigel was evaluated as described previously^24^. Briefly, ice-cold Matrigel (BD, Bedford, MA, USA) was layered in a 96-well plate and polymerization was allowed by incubating at 37 °C for 30 min. HUVECs (2 × 10^4^ cell/well) were plated onto the Matrigel layer in the culture medium for 6 h. The tubes formation was photographed using a digital camera from randomly chosen fields.

### Molecular docking

With the docking application GOLD-dock 5.0, the binding modes of the synthesized compounds to the active pocket of proteins were investigated. The crystal structure of AKT (PDB ID: 3O96) and mTOR (PDB ID: 4JT6) were used as protein receptor in molecular docking. The missing hydrogen atoms and residues were modeled in Tripos Sybyl 6.8.17. Atomic charges were taken as Kollman-united-atom for the receptor and Gasteiger–Marsili for the ligands. A genetic algorithm search performed the energy minimization during the validation of ligand binding to the receptor site. Ten docked conformations of each compound were generated after a reasonable number of evaluations. The conformation with highest GOLD score was then calculated to obtain the binding energy with TP enzyme by using protocol (MM-PBSA-BINDING ENERGY) in software Accelrys Discovery 3.0.

### Xenograft assay

Balb/c nude mice (6–8 week, 18–20 g) were purchased from Hunan SJA Laboratory Animal Co., Ltd. (Hunan, China). Then 100 μl/point of a 1:1 slurry containing Matrigel (BD Bioscience) and 5 × 10^6^ viable HepG2 cells were subcutaneously (S.C.) injected into the left flank of the nude mice. 10 days after cell injection, the average tumor volume reached about 100 mm^3^, mice were randomly divided into 4 groups (*n* = 6) and oral administration the following regimens every two days for 3 weeks: (1) saline; (2) ZJQ-24(20 mg/kg); (3) ZJQ-24 (40 mg/kg); (4) ZJQ-24 (60 mg/kg). Tumor volume and body weight were recorded every two days with a caliper (calculated volume = shortest diameter^2^ × longest diameter/2). After three weeks, mice were sacrificed and solid tumors were removed for further analyses. Animal studies were approved by Institutional Animal Care and Use Committee (IACUC) of the Kunming University of Science and Technology (KMUST) (Kunming, China), and were conducted in accordance with its recommendations and ethical regulations. All experimental protocols were approved by IACUC of KMUST. The mice were maintained under standard conditions according to the institutional guidelines for animal care.

### Cell culture and cell viability assay

The cell lines (HUVECs, MRC5, HepG2, HUH-7) were obtained from the American Type Culture Collection (ATCC, Manassas, VA). All cells were tested for mycoplasma contamination. The HUVECs were cultured in M200 medium and other cells were cultured in Dulbecco’s modified Eagle’s medium (DMEM) (Hyclone, Thermo Scientific) at 37°C in a humidified atmosphere of 5% CO_2_ with 10% FBS(GIBCO, Invitrogen, NY, USA). Cell viability was assessed using a 3-(4,5-dimethyl-2-thiazolyl)−2,5-diphenyl-2-H-tetrazolium bromide (MTT) assay. For HUVECs viability assay, cells were treated with VEGF (50 ng/ml) for 40 min except the control group. Then the medium was replaced with or without ZJQ-24 (1 μM, 5 μM, 10 μM, 20 μM, and 30 μM) and treated for 72 h. For other cells viability assay, cells were treated with ZJQ-24 in various concentrations (0.1 μM, 1 μM, 5 μM, 10 μM, and 50 μM) for 72 h. Then 25 μl MTT solution (5 mg/ml) was added to the medium, and the plates were incubated at 37°Cfor 4 h in a humidified atmosphere of 5% CO_2_. The formazan crystals formed by the mitochondrial reduction of MTT were solubilized in DMSO (150 μl/well) and the absorbance at 490 nm was measured using a microplate reader (Bio-Tek Inc., USA).

### Cell cycle and apoptotic cell death assay

For cell cycle analysis, HepG2 and HUH-7 cells were respectively seeded in a 6-well culture plate at 2 × 10^5^/well density, and allowed to attach overnight. Then, the cells were exposed to different concentrations of ZJQ-24(1 μM, 5 μM, 10 μM) for 24 h, and then were collected and fixed with cold 70% ethanol at 4 °C overnight. Following washing with PBS, cells were suspended in a staining solution containing 10 mg/ml RNase A, 400 mg/ml propidium iodide (PI) and 0.1% Triton-X at room temperature (RT) for 30 min. The stained cells were analyzed by flow cytometry.

Apoptotic cells were determined by Annexin V/PI staining analyses. Briefly, HepG2 and HUH-7 (2 × 10^5^ per/well) cells were respectively seeded into 6-well plates and treated with different concentrations of ZJQ-24(1 μM, 5 μM, 10 μM) for 24 h. The cells were then harvested and washed once with cold PBS. Cell surface levels of phosphatidylserine were quantitatively estimated using Annexin V-fluorescein isothio cyanate (FITC) and a PI apoptosis kit according to the manufacturer’s instructions (BD Biosciences, San Jose, CA, USA). The stained cells were analyzed by flow cytometry (Accuri C6, BD Biosciences) within 1 h.

### Samples of HCC

20 HCC patients samples were obtained from the First People’s Hospital of Yunnan Province. All patients signed an informed consent in accordance with the Declaration of Helsinki to provide an excess of tissues for research using Institutional Review Board approved protocols.

### Western blot analysis

The total cells were treated in lysis buffer [150 mM NaCl, 10 mM Tris (pH 7.2), 5 mM EDTA, 0.1% Triton X-100, 5% glycerol, and 2% SDS]. Each amount of the protein extracts were denatured by boiling at 95 °C for 10 min in sample buffer (Bio-Rad Laboratories, Inc., Hercules, CA, USA). The total proteins were separated by SDS-PAGE electrophoresis and transferred to a PVDF membrane. After blocking at room temperature for 1 h using PBST containing 10% BSA with gentle shaking, the membranes were incubated with primary antibodies (1:1000) overnight at 4 °C with gentle shaking, incubated with horseradish peroxidase-conjugated secondary antibodies (Santa Cruz Biotechnology, Inc.), and then visualized with the enhanced chemiluminescence (ECL) detection system (GE Healthcare, Piscataway, NJ, USA). All of the primary antibodies (mTOR, PDK1, PRAS40, 4EBP-1, P70S6K, S6, Raptor, Rictor, eIF4E, eIF4G, HIF-1α, Cyclin D1, C-myc, Bcl2, VEGF, PARP, Caspase-3, Tubulin, GAPDH and β-actin) were purchased from Cell Signaling Technology, Inc. (Danvers, MA, USA).

### Polymerase chain reaction (RT-PCR)

Real-time polymerase chain reaction (RT-PCR) analysis was performed as described previously^25^. Total RNA was extracted from control or ZJQ-24-treated cells by using TRIzol reagent (Invitrogen), and 1 μg total RNA was reverse-transcribed into cDNA using High Capacity cDNA Reverse Transcription Kit according to the manufacturer’s instructions (Applied Biosystems). The SYBR Green assay was used for qRT-PCR with the 7300 Real-Time PCR System (Applied Biosystems) and default cycling conditions. The following primers were used: Bcl-2 forward, 5′-ATGGGATCGTTGCCTTATGC-3′ and Bcl-2 reverse, 5′-CAGTCTACTTCCTCTGTGATGTTGT-3′; VEGF forward, 5′-AGTACATCTTCAAGCCATCCTG-3′ and VEGF reverse, 5′-TGCTCTATCTTTCTTTGGTCTGC-3′; c-myc forward, 5′-GTCAAGAGGCGAACACACAAC-3′ and c-myc reverse, 5′-TTGGACGGACAGGATGTATGC-3′; Cyclin-D1 forward, 5′-GCTGCGAAGTGGAAACCATC-3′ and Cyclin-D1 reverse, 5′-CCTCCTTCTGCACACATTTGAA-3′; β-actin forward, 5′-CTCCATCCTGGCCTCGCT-3′ and β-actin reverse, 5′-GCTGTCACCTTCACCGTTCC-3′. Real-Time System using a program consisting of 95 °C for 10 min, then 40 cycles of 95 °C for 15 s and 60 °C for 1 min. Data analysis was performed using the following equations: ΔC = C_t_(sample) − C_t_(endogenouscontrol);ΔΔC_t_ = ΔC_t_(sample) − C_t_(untreated); and fold change = 2−ΔΔC_t_.

### Co-immunoprecipitation assay

HepG2 cells were treated with different concentrations of ZJQ-24 (1 μM, 5 μM, 10 μM) for 24 h. After clarification of the lysates by centrifugation, the specific antibodies were added and complexes were allowed to form by incubating with rotation overnight at 4 °C. Slurry of protein A-Sepharose was then added and the incubation continued for 2 h. Immunoprecipitates captured with protein A-Sepharose were washed 3 times with RIPA buffer and analyzed by western blot as described above.

### Immunohistochemical analysis

Immunohistochemical analysis was performed as described previously^26^. Briefly, slides from formalin-fixed paraffin-embedded tissue blocks were deparaffinized and endogenous peroxidase activity was inhibited using H_2_O_2_. Samples were then stained using primary antibodies (1:50) at 4 °C overnight. The goat anti-rabbit or mouse IgG/horseradish peroxidase was applied as the secondary antibodies according to the standard protocols provided by the manufacturer, and followed by incubation of Vectastain ABC Kit (Vector Laboratories).The slides were examined under an inverted microscope at 200× magnification (Eclipse TS100, Nikon, Japan).

### Specificity kinase activity assay

The kinase activity assays were performed as previously described^27^. Briefly, all assays were carried out robotically at room temperature and were linear with respect to time and enzyme concentration under the conditions used. Assays were performed for 30 min using Multidrop Micro reagent dispensers (Thermo Electron Corporation, Waltham, MA, USA) in a 96-well format. Assays were stopped by addition of 5 μl EDTA (50 mM) and 2% CHAPS was transferred to a Lumitrac 200 plate and incubated for 30 min at room temperature before reading in a microplate reader (Bio-Tek Inc., USA).

### Statistical analysis

The experimental data were expressed as mean±standard deviation (SD). Statistical differences were evaluated using the two-tailed Student’s *t*-test and analysis of variance (ANOVA) followed by *q-* test, considered significant at *p < 0.05, **p < 0.01 or ***p < 0.001.

## Results

### Docking study and anti-angiogenesis effect of ZJQ-24

Although ZJQ-24 significantly downregulated the levels of p-VEGFR2 in HUVEC cells, the activity of VEGFR2 kinase was not prominent^23^. To clarify the functional role of ZJQ-24 in the antiangiogenic effect, we procceded to examine the interaction of ZJQ-24 with mTOR (PDB ID: 4JT6) and AKT(PDB ID: 3O96) proteins. All docking runs were applied using the GOLDdock 5.0 docking tools. The results of binding model are depicted in Fig. 1B. For mTOR protein, there was a special hydrogen-bonding formed which is oxygen atom of amide of ZJQ-24 with hydrogen atom of Tyr2225. Additional, there were also two hydrophobic binding site formed. For AKT protein, only one hydrogen-bonding was observed, and two hydrophobic binding site formed. Next, we invisigated the binding energies between ZJQ-24 and enzymes (mTOR, AKT, and VEGFR2, respectively) using protocol (MM-PBSA-BINDING ENERGY) in software Accelrys Discovery 3.0. As shown in Fig. 1C, the combination of ZJQ-24 and mTOR is the most stable with binding energy being −22.179 KJ/mol. And the binding energies for ZJQ24 with AKT and VEGFR2 are −20.875 KJ/mol and −8.8887 KJ/mol, respectively.

To further determine the antiangiogenic activity of ZJQ-24, tube formation assay was performed. The tube formation assay exhibited that the formation of capillary-like tubes was inhibited significantly in the presence of ZJQ-24 in a concentration-dependent manner (Fig. 1D). These results further indicated that ZJQ-24 could significantly inhibit angiogenesis which was consistent with previous study^23^.

### Anti-hepatocellular carcinoma effects of ZJQ-24 in vivo and in vitro

**Fig. 2:**
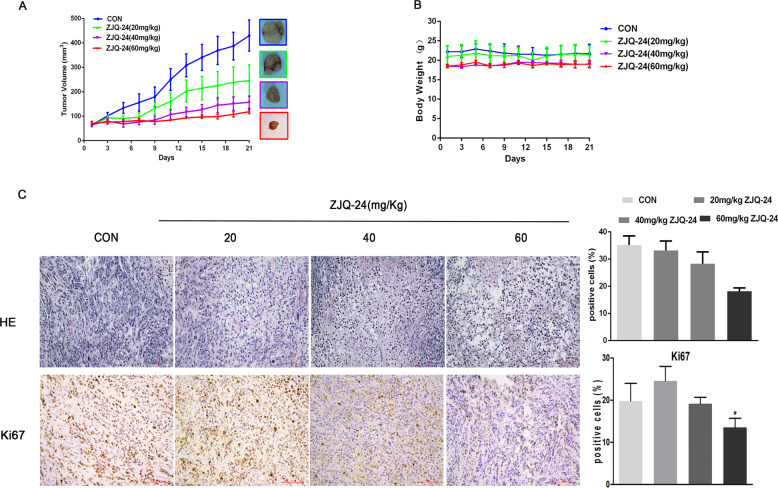
Anti-hepatocellular carcinoma effects of ZJQ-24 in vivo. The HepG2 cells were injected subcutaneously into nude mice and treated with ZJQ-24 (20 mg/kg), ZJQ-24 (40 mg/kg), ZJQ-24 (60 mg/kg) every two days for 3 weeks. **A** Tumor volumes was monitored over 21 day period, presented as mean ± SD. **B** The bodyweight of HepG2 xenograft mouse. **C** H&E staining and immunohistochemical staining were performed to analyze the number of tumor cells and the expression levels of Ki67 in tumor tissues.

To assess whether ZJQ-24 can inhibit the growth of hepatocellular carcinoma, a hepatocellular carcinoma xenograft model was first established by inoculating nude mice with HepG2 cells. As shown in Fig. 2A, ZJQ-24 significantly suppressed the growth of HepG2 tumors, and the relative tumor volume in ZJQ-24 (60 mg/kg) group reduced 72.6 % compared with control group (*p* < 0.001). Most encouragingly, ZJQ-24 did not affect mice body weight (Fig. 2B) or cause other observable side effect compared with control, suggesting that ZJQ-24 exhibited little toxicity and few side effects.

In addition, the H&E staining of tumor tissue further verified this conclusion, compared with control group, the 60 mg/kg ZJQ-24 treated group showed a significant reduction in the number of tumor cells, partial degeneration and necrosis of tumor cells and a large area of vacuoles in the tumor tissue. Immunohistochemistry of tumor tissues also revealed that the expression level of nuclear protein Ki67 which was strongly associated with tumor cell proliferation and growth^28^ was significantly decreased in samples treated with ZJQ-24 (Fig. 2C).

Furthermore, the MTT assay showed that ZJQ-24 could significantly inhibit the proliferative of hepatocellular carcinoma cells, such as HepG2 and HUH-7 cells. As shown in Table 1, the IC_50_ values of ZJQ-24 were 3.54 ± 0.38 μM for HepG2, and 6.59 ± 0.32 μM for HUH-7 cells. In contrast, concentrations of ZJQ-24 from 0 to 100 μM caused no obvious toxic effect on the normal human embryonic lung fibroblasts cell line MRC-5. In addition, the cisplatin (DDP) is one of the clinical drugs used to treat HCC, so we used it as a positive control to treat HepG2, HUH-7, and MRC-5 cells. And the results showed that IC50 values of DDP were 5.41 ± 0.08 μM for HepG2, 9.23 ± 0.83 μM for HUH-7 cells and 8.93 ± 0.39 μM for MRC-5 cells. Thus, ZJQ-24 had a better inhibitory effect on HCC than DDP and also showed a high selectivity in hepatocellular carcinoma cells. These results strongly suggested that ZJQ-24 could significantly inhibit hepatocellular carcinoma in vivo and in vitro.

**Table 1:** IC_50_ values of ZJQ-24 and DDP on the growth inhibition of HCC and MRC-5.

	Cell lines IC_50_ (μM)			
Compound	HepG2	HUH-7		MRC5
ZJQ-24	3.54 ± 0.38		6.59 ± 0.32	>100
DDP	5.41 ± 0.08		9.23 ± 0.83	8.93 ± 0.39

The growth inhibitory activity, determined as IC_50_ values for each cell line by 72 h MTT assay. Data represent the mean values of three independent experiments.

### ZJQ-24 induces cell cycle arrest and apoptosis in HCC cells

**Fig. 3:**
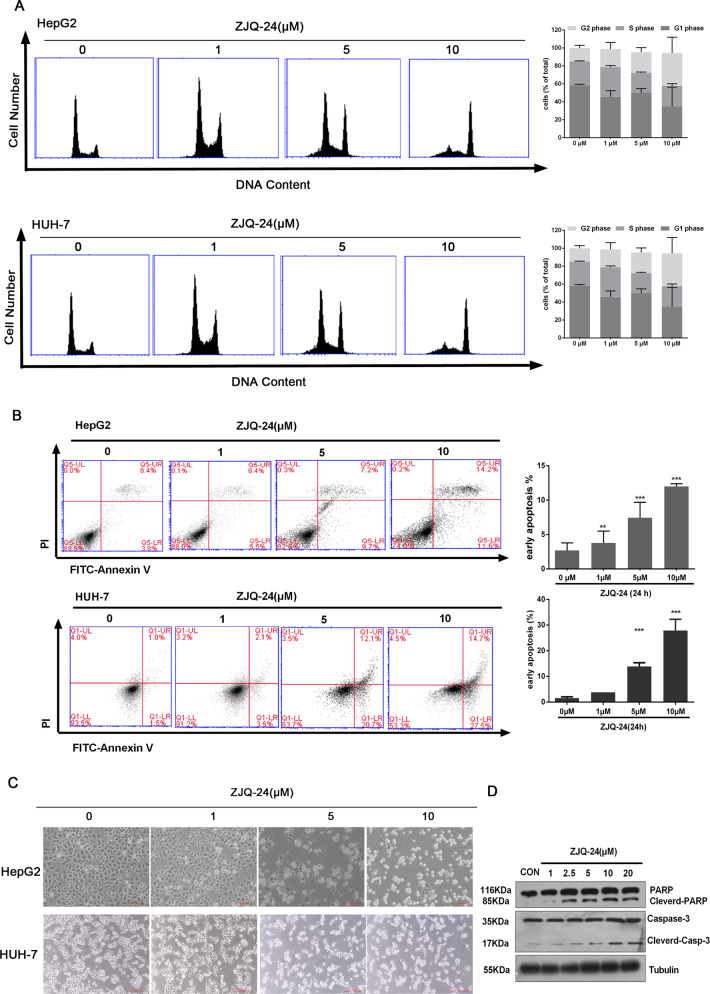
ZJQ-24 induces cell cycle arrest and apoptosis in HCC cells. **A** Representative DNA histograms and quantitative analysis of cell cycle in HepG2 and HUH-7 cells after ZJQ24 treatment. **B** Induction of apoptosis and quantitative assessment of early apoptotic cells by ZJQ-24 treatment in HepG2 and HUH-7 cells. **C** The cellular morphology of HepG2 and HUH-7 cells after ZJQ-24 treatment. **D** Detection of apoptosis proteins following ZJQ-24 treatment for 24 h, lysates from HepG2 samples were subjected to SDS-PAGE and probed with antibody to PARP and caspase-3. **P < 0.01, ***P < 0.001 vs. control group.

To identify the functional role of ZJQ-24 in the anti-hepatocellular carcinoma effect, we explored the effect of ZJQ-24 on cell cycle progression. Flow cytometry analysis revealed that after 10 μM ZJQ-24 treatment for 24 h, compared with the control, the number of HepG2 and HUH-7 cells in G_2_/M phase were increased and the number of cells in G_1_ phase were decreased (Fig. 3A). These results suggest that ZJQ-24 could induce cell cycle arrest.

Furthermore, we investigated the effects of ZJQ-24 on cell apoptosis through Annexin-V/PI double staining assay. The flow cytometry results showed that the proportion of early apoptotic HepG2 and HUH-7 cells increased significantly in a dose-dependent manner after treatment of ZJQ-24 for 24 h (Fig. 3B). Moreover, a typical apoptotic morphological changes were observed by microscope, such as condended chromatin, fragmented nuclei and apoptotic bodies (Fig. 3C). The expression of apoptosis-related proteins was further investigated via western blotting. ZJQ-24 treatment resulted in upregulation of cleavage caspase-3 and PARP in a dose-dependent manner (Fig. 3D). Together, these results indicate that ZJQ-24 induces G_2_/M phase arrest and apoptosis in HCC cells.

### ZJQ-24 promotes cell death via inhibition of AKT/mTOR pathway in vivo and in vitro

**Fig. 4:**
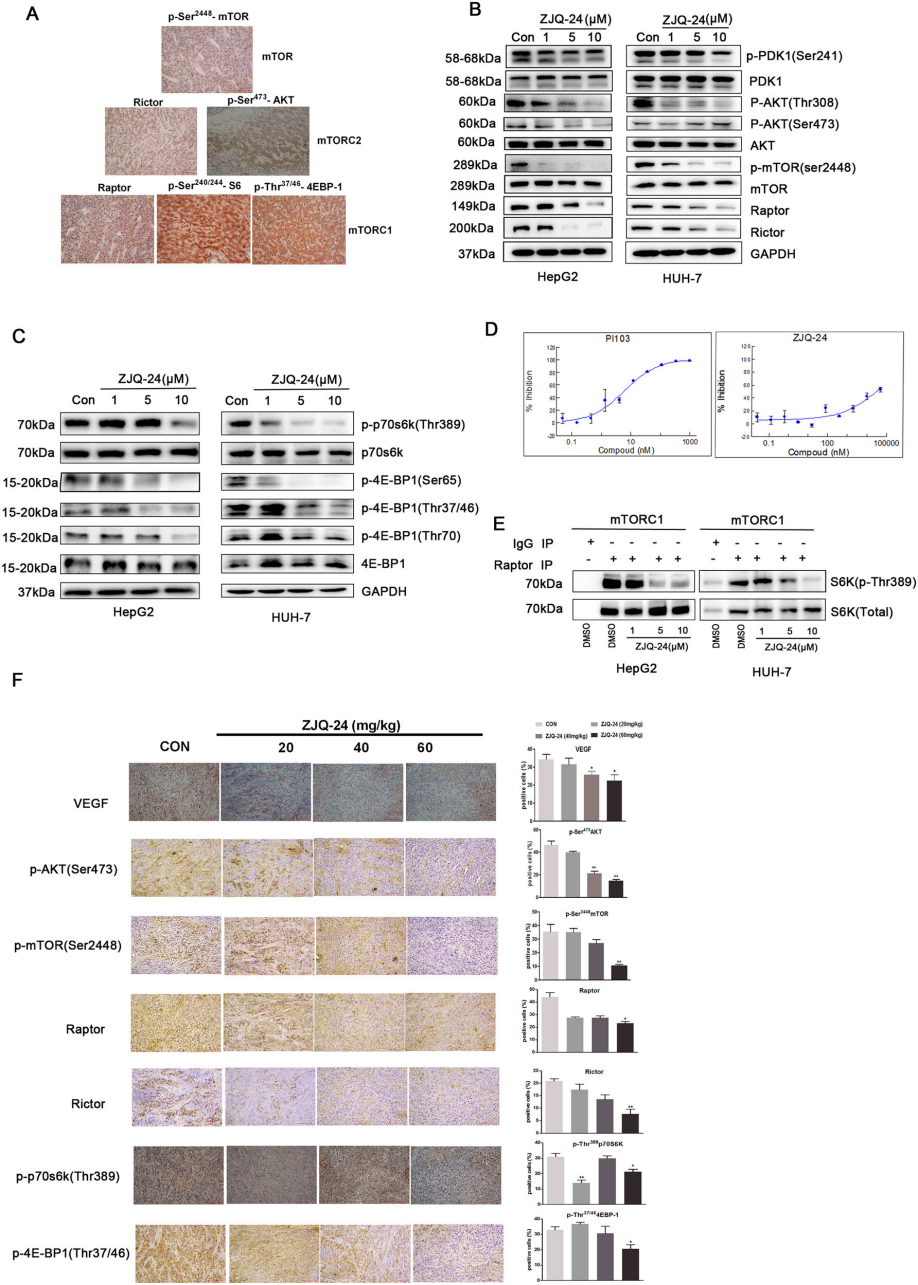
Effects of ZJQ-24 on the AKT/mTOR pathway in vivo and in vitro. **A** Expressions of mTORC1 and mTORC2 in clinical HCC samples. HCC samples (*n* = 20) were stained using antibodies to p-mTOR(Ser^2448^), Rictor, Raptor, and targets downstream of mTORC1 (p-4EBP1(Thr^37/46^), p-S6(Ser^240/244^)) and mTORC2 (p-Akt(Ser^473^)). **B** ZIQ-24 inhibited PI3K/AKT/mTOR signaling pathway proteins. **C** ZIQ-24 inhibited the expression of substrate of mTORC1. **D** ZIQ-24 inhibited the lipid kinases activity of mTORC. **E** ZJQ-24 inhibited the endogenous kinases activity of mTORC1. HepG2 and HUH-7 cells extracts were subjected to immunoprecipitation (IP) with pre-immune (IgG) or anti-Raptor. The immunoprecipitates were incubated with dephosphorylated GST–S6K1 in the presence of the indicated concentrations of ZJQ-24 or DMSO. **F** The expressions of VEGF, p-AKT (Ser^473^), p-mTOR (Ser^2448^), Raptor, Rictor, p-4EBP-1 (Thr^37/46^), and p-P70S6K (Thr^389^) were measured by immunohistochemistry with tumor tissues.

The previous studies have reported that PI3K/AKT/mTOR signaling was significantly activated in HCC^29^. However, whether both mTORC1 and mTORC2 are activated in clinical HCC samples have not been detected. As indicated in Fig. 4A, expression of raptor (a component of mTORC1) and the mTORC1 downstream targets phospho-S6 (Ser^240/244^) and phospho-4EBP-1 (Thr37/46) were significantly demonstrated, accompanying with phosphorylation of mTOR at Ser^2448^ in clinical HCC samples (*n* = 20). Meanwhile, expression of rictor (a component of mTORC2) and the mTORC2 substrate phospho-AKT (Ser^473^) was also observed. These observations further proved a potential rational hypothesis for targeting both mTORC1 and mTORC2 complexes in HCC.

Given the inhibition of proliferation in HepG2 cells treated with ZJQ-24 is associated with the down-regulation of AKT/mTOR pathway, we next investigated the proteins expression of AKT/mTOR pathway with ZJQ-24 treatment for 24 h. As shown in Fig. 4B, 10 μM ZJQ-24 strongly decreased the proteins of AKT/mTOR pathway, including the phospho-PDK1(Ser^241^), phospho-AKT (Thr^308^/Ser^473^), phospho-mTOR (Ser^2448^), Raptor and Rictor in HepG2 and HUH-7 cells. In addition, the downstream molecules of mTORC1, including phospho-p70S6K (Thr^389^) and phospho-4EBP-1 (Ser65, Thr37/46, Thr^70^) were also downregulated (Fig. 4C). These results suggested that ZJQ-24 might be an inhibitor of mTORC1.

To evaluate the specificity of ZJQ-24, we studied the effect of ZJQ-24 at ATP concentrations, which approximate to the K_m_ constant for ATP. As shown in Fig. 4D, ZJQ-24 inhibited the mTOR lipid kinase activity (EC_50_ = 40.645 μM). However, the inhibition effect was not strongly as the PI103, which is a special inhibitor of PI3K. Therefore, we used Co-IP assay to identify the inhibition activity of endogenous immunoprecipitated mTORC1, assayed employing S6K1 as substrate. At 10 μM ZJQ-24, which diminished the binding of phospho-p70S6K(Thr^389^) with mTORC1(Raptor) (Fig. 4E). It suggested that ZJQ-24 completely suppressed the kinase activity of mTORC1.

To further elucidate the inhibition effect of ZJQ-24 on AKT/mTOR pathway in vivo, we examined the expression level of AKT/mTOR pathway. Consistent results observed in vitro that ZJQ-24 profoundly inhibited AKT/mTOR pathway, as evidenced by the reduction of p-AKT(Ser^473^), p-mTOR(Ser^2448^), Raptor, Rictor p-4EBP1(Thr^37/46^), and p-S6(Ser^240/244^) expression. In addition, the antiangiogenic effect of ZJQ-24 was further evidenced by VEGF reduction (Fig. 4F).

Taken together, these data suggest that ZJQ-24 inhibited AKT/mTOR pathway in vivo and in vitro without inducing AKT feedback activation.

### ZJQ-24 inhibits AKT feedback activation through active JNK/IRS-1 in HCC cells

**Fig. 5:**
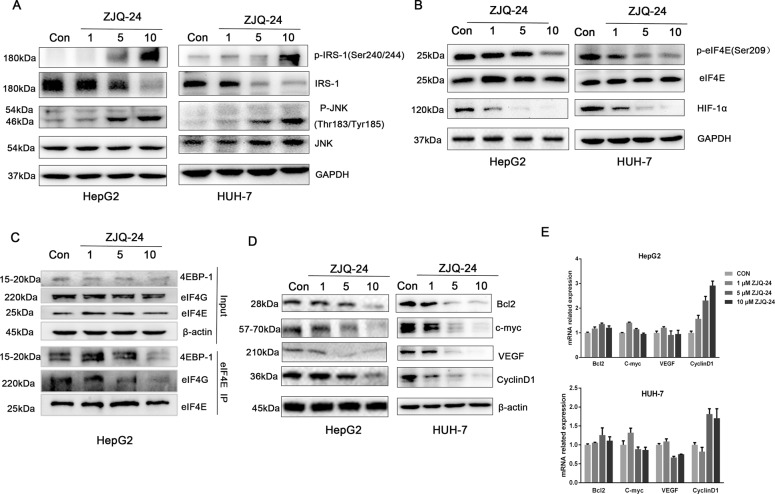
Effects of ZJQ-24 on JNK/IRS-1 pathway and cap-dependent translation in HCC cells. **A** The expressions of IRS-1 and JNK by ZJQ-24 treatment. **B** The expressions of eIF4E and HIF-1α by ZJQ-24 treatment. **C** Effect of ZJQ24 on eIF4E complex. **D** The expressions of Bcl-2, C-myc, VEGF, Cyclin D1, and β-actin by ZJQ-24 treatment. **E** mRNA expression of Bcl-2, C-myc, VEGF, Cyclin D1 by ZJQ-24 treatment.

The above studies demonstrated that the inhibition of kinase activity of mTORC1 by ZJQ-24 does not induce the AKT feedback activation which is showed as mTORC2 and phospho-AKT (Ser^473^) down-regulation. The mTOR inhibitors rapamycin only block the mTORC1 has been reported to induce AKT phosphorylation through p70S6K and subsequent insulin receptor substrate 1 (IRS-1) hypophosphorylation^30^. The phosphorylation of IRS-1 promotes IRS-1 degration and reduces its abundance, leading to decreased activity of PI3K and Akt^31^. We hypothesized that the inhibition of phospho-AKT(Ser^473^) is associated with the active of phosphorylation of IRS-1. Therefore, we next investigated the expression of phospho-IRS-1(Ser^240/244^).Unexpectedly, we found that inhibition of phospho-p70S6K by ZJQ-24 did not block IRS-1 phosphorylation (Fig. 5A). We speculate that ZJQ-24 active IRS-1 phosphorylation through other kinases. C-Jun N-terminal kinase (JNK) has been reported to phosphorylate the IRS-1, leading to an inhibition of downstream signaling pathways activated by insulin, including Akt/mTOR^32^. Hence, we examined the level of phospho- JNK (Thr^183^/Tyr^185^) after ZJQ-24 treatment. AS shown in Fig. 5A, ZJQ-24 enhanced the expression of phospho- JNK (Thr^183^/Tyr^185^) in a dose-dependent manner. These results suggested that ZJQ-24 inhibits Akt phosphorylation at Ser473 through active JNK to phosphorylate IRS-1 in HCC cells.

### ZJQ-24 inhibits cap-dependent translation in HCC cells

It is known that hypo-phosphorylated 4EBP-1 disrupts eIF4F complex formation by binding competitively to eIF4E and inhibits cap-dependent translation^33^. To determine whether inhibition of phospho-4EBP-1 by ZJQ-24 can also suppress cap-dependent translation in HCC cells. We first examined the expression of eIF4E. Clearly, 10 μM ZJQ-24 significantly inhibited the expression of phospho-eIF4E Ser^209^ and HIF-1α, which was the downstream gene of eIF4E in a dose-dependent manner (Fig. 5B). As expected, ZJQ-24 (10 μM) disrupted the interaction of eIF4G and eIF4E, and also inhibited the binding of 4EBP-1 and eIF4E (Fig. 5C).

To further confirm this result, we evaluated the effect of ZJQ-24 on cap-dependent translation and found that ZJQ-24 significantly decreased the protein level of Bcl-2, c-myc, VEGF, and Cyclin D1 (all of which are oncogenic proteins encoded by “weak” mRNAs) but not β-actin (which is a classic housekeeping protein and is encoded by a “strong” mRNA)^34^ (Fig. 5D). It is noteworthy that ZJQ-24 did not affect the mRNA level of these oncogenic proteins (Fig. 5E). These results illustrated that ZJQ-24 could down-regulate weak mRNAs translation which was cap-dependent but had no effect on strong mRNAs translation which was cap-independent. The above results strongly suggested that ZJQ-24 inhibited AKT/mTOR and cap-dependent translation collectively trigger cell cycle arrest and apoptosis to promote cell death.

## Discussion

Our previous study had synthesized a novel series of indole-2-carbohydrazide compounds as VEGFR2 tyrosine kinase inhibitor^23^. However, the potential of an active compound ZJQ-24 to kill hepatocellular carcinoma cells and the underlying mechanism have not yet been elucidated. In a xenograft nude mouse model, we observed that ZJQ-24 treatment significantly suppressed HCC growth in a dose-dependent manner but had not effect on the mice weight, indicating that the compound is well tolerated. H&E staining and immunohistochemistry of tumor tissues showed that ZJQ-24-treated significantly decreased the tumor cells and Ki67 expression. In vitro, ZJQ-24 significantly suppressed the proliferation of HepG2 and HUH-7 cells after incubation for 72 h. Moreover, there was no significant cytotoxic effect of ZJQ-24 on MRC-5 cells. Therefore, ZJQ-24 has high anti-cancer effect, low toxicity, and few side effects.

The predominant molecular mechanism underlying the anti-cancer activity of ZJQ-24 was associated with angiogenesis inhibition, G_2_/M cycle arrest and cell apoptosis. First, HCC is highly vascular tumor with a strong angiogenesis-inducing ability. The tube formation assay showed that ZJQ-24 inhibited the formation of capillary-like tubes. Second, cell cycle dysregulation is a character of tumor cells, and the induction of cell cycle arrest is an potential target for anti-cancer^35^. Flow cytometry analysis showed that ZJQ-24 induced G_2_/M phase arrest in HepG2 cells. When cells experience DNA damage, the cell cycle check point will be active through tumor suppressor to repair these mistakes. If the damage is so severe that the cells cannot be repaired, cells will go to apoptosis. Third, apoptosis is a process by the activation of caspases, which regulate the exogenous death receptor pathway and the endogenous mitochondrial pathway. Annexin-V/PI double staining assay showed that the population of apoptotic cells increased significantly after ZJQ-24 treatment. Western blot analysis showed that PARA and caspase-3 increased in a dose-dependent manner, further implying that mitochondrial dysfunction was induced by ZJQ-24 in HepG2 cells. Therefore, ZJQ-24 induced caspase-dependent apoptosis via intrinsic pathway in HepG2 cells.

The PI3K/AKT/mTOR pathway is involved in the regulation of cancer cellular processes, including cell proliferation, migration, invasion, and survival^36^. The activation of mTOR, an important downstream target of AKT, can promote angiogenesis by increasing VEGF expression or inducing HIF-1α-dependent gene expression in tumor cells^37,38^. Dysregulation of mTOR signaling often occurs in a variety of cancer, rendering it a promising target in cancer therapy. Our molecular modeling study showed that ZJQ-24 bound to mTOR and AKT proteins. For mTOR protein, there was a special hydrogen-bonding formed which is oxygen atom of amide of ZJQ-24 with hydrogen atom of Tyr2225, there were also two hydrophobic binding site formed additionally. For AKT protein one hydrogen-bonding was observed, and two hydrophobic binding sites formed. The binding model suggests that compound ZJQ-24 is a potential inhibitor of mTOR and Akt.

It has been observed that PI3K/AKT/mTOR pathway is activated in HCC, indeed, increased mTORC1/2 and downstream targets AKT, S6 and 4EBP-1 were activated in HCC as determined based on Rictor, Raptor over-expressions and enhanced Akt phosphorylation (Ser^473^). The efficacy of rapamycin and its analogs (rapalogs) is limited, which all sterically inhibit mTORC1. As mTORC1 suppression activates negative feedback loops to promote cell survival, rapamycin and rapalogs incompletely inhibit mTOR signallling^39^. We show here that ZJQ-24 is an effective inhibitor of mTORC1 which is supported by the decrease of both S6K and 4EBP-1 phosphorylation. In contrast to rapamycin-induced feedback activation of AKT with limited inhibitory activity on mTORC2, ZJQ-24 would be the mTORC2 blockade leading to decrease of phosphorylation of AKT at Ser473. We further noticed that the expression of Akt/mTOR pathway in tumor tissues were downregulated in a xenograft nude mouse mode. These observations further confirmed inhibitory effects of ZJQ-24 on tumor growth are associated with the down-regulate of mTOR pathway.

In addition, phosphorylation of Thr308, a PDK1 phosphorylation site was also inhibited by ZJQ-24. It has been reported that mTORC1/p70S6K represses upstream PI3K signaling through phosphorylation of IRS-1^40^. The phosphorylation of IRS-1 promotes IRS-1 degradation and resudes its abundance, leading to inhibition of PI3K/AKT. However, we demonstrated that ZJQ-24 suppresses p70S6K did not decrease the phosphorylation of IRS-1. Hence it is clear that inhibition of p70S6K by ZJQ-24 does not mimic the activation of AKT through decreasing the phosphorylation of IRS-1. Therefore, we further analysis the activity of JNK which has been reported to phosphorylate the IRS-1. As predicted, ZJQ-24 potently increased phosphorylation of JNK on Thr^183^/Tyr^185^. These collective results suggested that ZJQ-24 can inhibit AKT/mTOR pathway without inducing feedback activation of AKT by active JNK/IRS-1 in HCC cells.

mTORC1 has been shown to regulate cap-dependent translation. To the extent that mTOR and 4EBP-1 down-regulation contribute to ZJQ-24-induced anti-angiogenesis and proliferation inhibition, one would predict that eIF4E expression and protein translation might be inhibited. The expression of eIF4E which was one polypeptide of the eIF4F complex and played a critical role in the transformation of malignant status and maintenance of transformed phenotypes^41^. In addition, high-level eIF4E has been identified to relate with the progression of tumor and low survival in several human cancers, such as breast, colon and lung cancers^42–44^. Our results showed that phospho-eIF4E Ser^209^ was decreased in response to ZJQ-24 treatment. As eIF4E is involved in cap-dependent translation, we further investigated the effect of ZJQ-24 on mRNA translation and found that ZJQ-24 effectively suppressed the translation of weak mRNA (e.g. VEGF, C-myc, Cyciln D1, and Bcl-2), while has no effect on strong mRNA translation (e.g. β-actin), indicating that ZJQ-24 inhibits cap-dependent translation. Previous studies have reported that eIF4E is phosphorylated on a unique site (Ser^209^, human sequence) by the MAPK-interacting protein kinases MNK, which are activated by Erk signaling. This process is dependent on eIF4G binding to eIF4E^45^. Our results showed that ZJQ-24 inhibited the binding of eIF4G and 4EBP-1to eIF4E. Taken together, the results indicated that ZJQ-24 inhibited the cap-dependent translation in HCC cells.

**Fig. 6:**
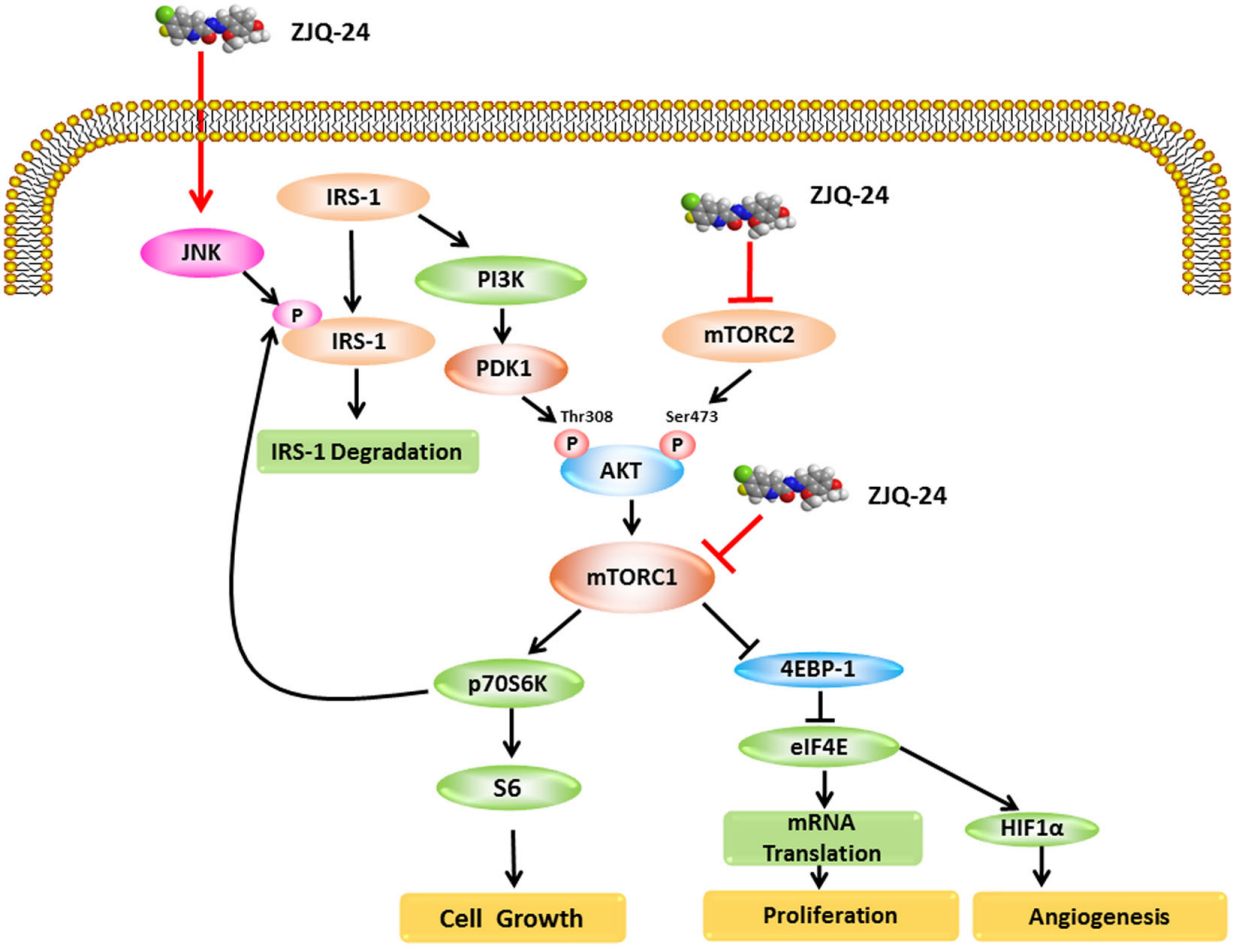
Schematic representation of the hypothesized molecular mechanism underlying the anti-cancer activity of ZJQ-24. The PI3K/AKT/mTOR and JNK/IRS-1 signaling pathway is involved in ZJQ-24-induced apoptosis and cap-dependent translation in Hepatocellular Carcinoma.

In summary, as shown in Fig. 6, we have provided the evidence that ZJQ-24 exhibits high anti-cancer activity, low toxicity, and few side effects in HCC and suppresses cell proliferation via G2/M phase arrest and cell apoptosis. We also confirmed that ZJQ-24 inhibits tumor growth and induces cell apoptosis through inhibition the kinase activity of mTORC1 without activation of AKT, which is associated with JNK/IRS-1 activation. Moreover, it inhibits angiogenesis and induces apoptosis also via inhibition the cap-dependent translation which regulated by mTORC1 signaling and eIF4F complex. This compelling evidences not only provide a target of HCC therapy but also provide insight to better understand the underlying mechanisms of the anti-cancer effect ZJQ-24.
