# Correction to: Indole hydrazide compound ZJQ-24 inhibits angiogenesis and induces apoptosis cell death through abrogation of AKT/mTOR pathway in hepatocellular carcinoma

**DOI:** 10.1038/s41419-021-03388-2

**Published:** 2021-01-25

**Authors:** Jing Liu, Ying Liu, Jianqiang Zhang, Dan Liu, Yafeng Bao, Tianxing Chen, Tao Tang, Jun Lin, Ying Luo, Yi Jin, Jihong Zhang

**Affiliations:** 1 Laboratory of Molecular Genetics of Aging and Tumor, Medical School, Kunming University of Science and Technology, Kunming, 650500 PR China; 2 Key Laboratory of Medicinal Chemistry for Natural Resource, Ministry of Education, School of Chemical Science and Technology, Yunnan University, Kunming, 650091 PR China; 3 College of Biology and Chemistry, Key Laboratory of Subtropical Medicinal Edible Resources Development and Utilization in Yunnan Province, Puer University, Puer, 665000 Yunnan China; 4 Pathology Department, The First People’s Hospital of Yunnan Province, Kunming, 650032 Yunnan PR China; 5 Guizhou Provincial Key Laboratory of Pathogenesis & Drug Development on Common Chronic Diseases, School of Basic Medicine, Guizhou Medical University, Guiyang, 550000 Guizhou China

Correction to: *Cell Death & Disease*

10.1038/s41419-020-03108-2 published online 28 October 2020

The original version of this article unfortunately contained a mistake in Fig. 3A. The correct figure can be found below. The authors apologize for the mistake. The original article has been corrected.

**Figure:**
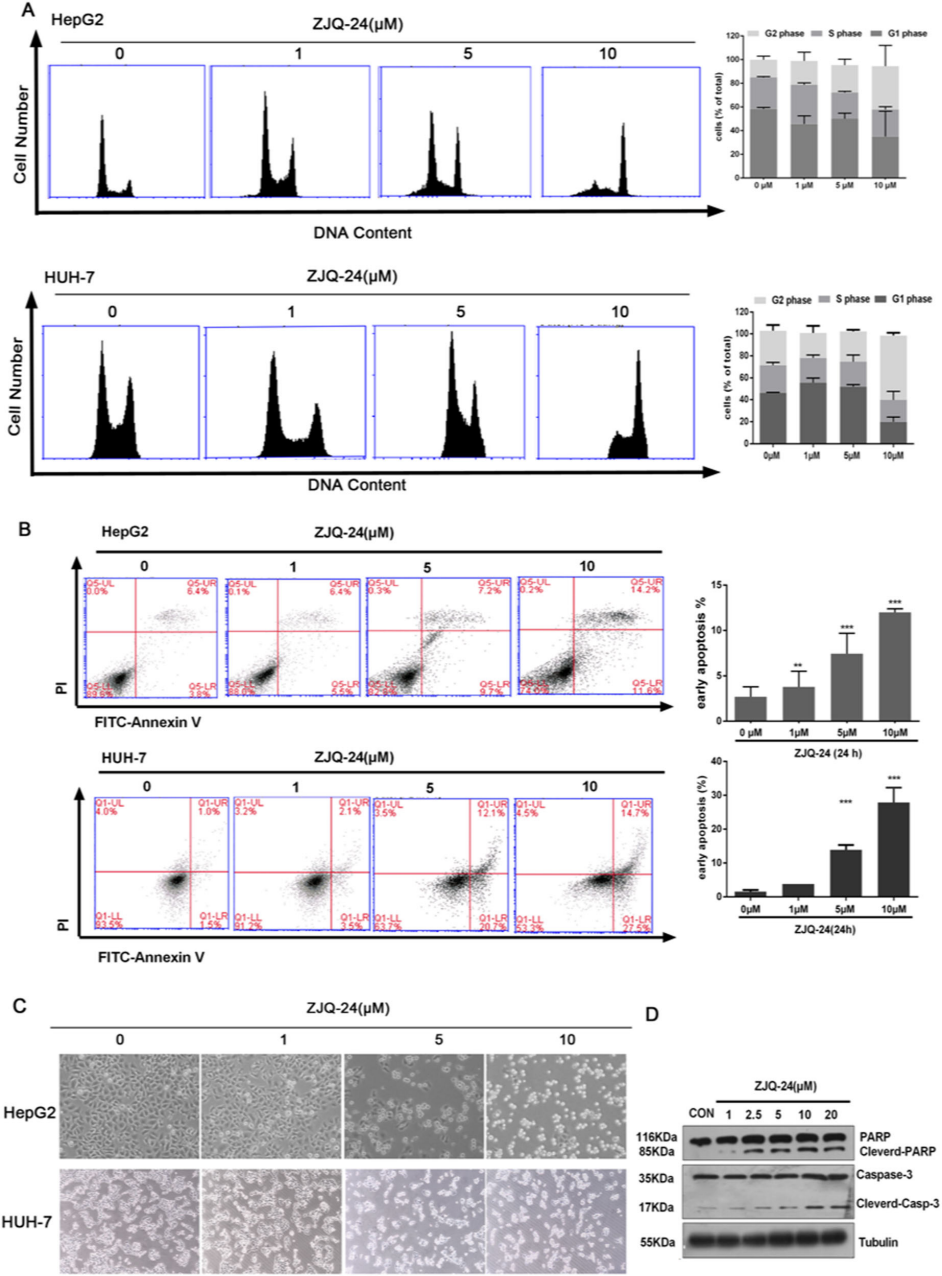
Fig. 3

